# CSSSCL: a python package that uses combined sequence similarity scores for accurate taxonomic classification of long and short sequence reads

**DOI:** 10.1093/bioinformatics/btv587

**Published:** 2015-10-09

**Authors:** Ivan Borozan, Vincent Ferretti

**Affiliations:** Informatics and Bio-computing, Ontario Institute for Cancer Research, MaRS Centre, 661 University Avenue, Suite 510, Toronto, Ontario, Canada

## Abstract

**Summary:** Sequence comparison of genetic material between known and unknown organisms plays a crucial role in genomics, metagenomics and phylogenetic analysis. The emerging long-read sequencing technologies can now produce reads of tens of kilobases in length that promise a more accurate assessment of their origin. To facilitate the classification of long and short DNA sequences, we have developed a Python package that implements a new sequence classification model that we have demonstrated to improve the classification accuracy when compared with other state of the art classification methods. For the purpose of validation, and to demonstrate its usefulness, we test the combined sequence similarity score classifier (CSSSCL) using three different datasets, including a metagenomic dataset composed of short reads.

**Availability and implementation:** Package’s source code and test datasets are available under the GPLv3 license at https://github.com/oicr-ibc/cssscl.

**Contact:** ivan.borozan@oicr.on.ca

**Supplementary information:** Supplementary data are available at *Bioinformatics* online.

## 1 Introduction

One important problem in computational molecular biology is the correct classification of unknown DNA sequences, given a database of sequences of known origin. Next-generation sequencing technologies have dramatically accelerated the study of microbial and viral genomes with the promise of uncovering thousands of previously unknown species. These high-throughput studies have, however, produced mostly short-read data (i.e. reads between 100 and 400 bp in length) that present a great challenge for phylogenetic classification (and other related metagenomic analysis (Brady and Salzberg, 2009; Rosen *et* *al.*, 2011; Wood and Salzberg, 2014) or automated genome assemblies (Koren and Phillippy, 2015). Emerging long read sequencing technologies such as PacBio RS and Oxford Nanopore MinION can already generate sequences over tens of kb in length that for the first time allow highly accurate automated assemblies of finished bacterial genomes (Koren and Phillippy, 2015; Loman *et* *al.*, 2015). In this article, we introduce the CSSSCL package for taxonomic classification of DNA sequences that implements the classification model presented in our recent paper (Borozan *et* *al.*, 2015), we describe its features and show its effectiveness at classifying sequences using three different datasets.

## 2 Package description

The CSSSCL package consists of two modules (i) the build_dbs module that creates all the necessary databases and (ii) the *classify* module that performs the classification.

### 2.1 Database creation

Before sequence classification can be performed, the user needs first to specify the collection of reference genomes composing the training set. Using sequences in the training set, the build_dbs module then creates three different databases (i) the BLAST database (using the blast algorithm), (ii) the kmer database [using the Jellyfish (Marais and Kingsford, 2011) multi-threaded k-mer counter] and (iii) the compression database (using the multi-threaded plzip compression algorithm). During this stage, the taxon information (from the NCBI taxonomy flat files) for each sequence is stored in the CSSSCL’s MongoDB a scalable, high-performance, open source document-oriented database allowing for fast retrieval and analysis of taxonomic information.

### 2.2 Sequence classification

The *classify* module classifies sequences in the test set using the combined sequence similarity scores (CSSSs) (as described in Borozan *et* *al.*, 2015) calculated based on the information stored in its pre-computed databases. The four measures implemented in the package are (i) the BLAST (BLASTN or MEGABLAST) -based measure (expressed in terms of the BLAST bit scores), (ii) the Euclidean distance (based on the relative abundance of kmers in each sequence), (iii) the Jensen Shannon Divergence (based on the relative abundance of kmers in each sequence) and (iv) the compression-based measure. The *classify* module allows users to specify one of the taxonomic levels (such as *species, genus, family, order, class or phylum*) at which the classification of sequences is to be performed. Prior to performing the classification, the module finds optimum values for its parameters [such as the optimum k-mer size and removes sequence similarity measures with the low predictive power (Borozan *et* *al.*, 2015)] based on the information obtained from the sequences in the training set and provides an estimate of the overall accuracy with which sequences are to be classified using a leave-one-out cross-validation procedure. Note that the module also allows users to specify which measures should be used by the model prior to the optimization phase. Finally, the module assigns the taxonomic label to each sequence in the test set by using the nearest neighbor algorithm and the CSSSs (Borozan *et* *al.*, 2015). CSSSCL is written in Python, is fully parallelized and should run on most UNIX-like systems.

## 3 Results

We use three different datasets (one viral and two bacterial) to demonstrate the ability of the CSSSCL program to accurately classify DNA sequences. The first dataset consists of viral nucleotide sequences that due to their considerable variability are expected to pose a greater challenge to most phylogenic classification algorithms. The entire set of viral genomes was downloaded from the NCBI RefSeq database. From a total of 5808 viral genomes, we selected 263 different genera with at least three different sequence entries per taxon label, producing a total of 3917 different complete viral sequences. This set of sequences was then split into two using the 2/3 (training) and 1/3 (test) splits (we also required that the training set contains at least two entries per taxon label at the genus level). The training and test sets produced in this way contained, respectively, 2900 and 1017 viral sequences. The second dataset consists of bacterial nucleotide sequences (Bacterial dataset I). The entire set of 5242 bacterial sequences was downloaded from the NCBI RefSeq database containing 2647 RefSeq complete genomes. From the entire set, we selected 277 different genera with at least three different sequence entries per taxon label, producing a total of 4601 different microbial sequences. These sequences were then split into the training and test sets (as explained above) containing, respectively, 3420 and 1181 bacterial sequences. To demonstrate that our program can also classify short reads, we use the metagenomic dataset Bacterial dataset II (MiSeq) introduced in Kraken (Wood and Salzberg, 2014) consisting of 10 000 reads with an average read length of 156 bp (the training set for this dataset is composed of the entire set of 5242 bacterial sequences mentioned above). In Table 1, we compare the performance of CSSSCL to two other classifiers namely NBC (Rosen *et* *al.*, 2011) and Kraken using the identical training and test sets. We chose NBC for its high accuracy and for being the most sensitive metagenomics classifier according to Bazinet and Cummings (2012) and Kraken for being currently the fastest metagenomics classifier according to Wood and Salzberg (2014). The results presented in Table 1 show that CSSSCL achieves higher precision and recall (or sensitivity) than either NBC or Kraken when classifying viral genomes and higher recall (or sensitivity) when classifying bacterial genomes. For short reads, CSSSCL outperforms in recall/sensitivity both NBC, Kraken and Kraken-GB (Sensitivity: 86.23 as presented in Wood and Salzberg, 2014) and achieves identical high precision as Kraken but a slightly lower precision than Kraken-GB (Precision: 98.84 as presented in Wood and Salzberg, 2014) that uses a much larger database.

**Table 1.** The classification performance across three datasets obtained with CSSSCL, NBC (Rosen *et al.*, 2011) and Kraken (Wood and Salzberg, 2014)

Classifiers	Viral (precision, recall, [gp/min, RAM (GB)], time_db (h:m), time_cl (h:m))	Bacterial I (precision, recall, [gp/min, RAM (GB)], time_db (h:m), time_cl (h:m))	Bacterial II (precision, recall, [rp/min, RAM (GB)], time_db (h:m), time_cl (h:m))
CSSSCL(blast, kmers, compression)	95.0, 94.0, [2, 4], 12 h 34 m, 7 h 41 m	NA*	NA
CSSSCL(blast, kmers)	91.0, 90.0, [254, 4], 1 h 23 m, 0 h 4 m	87.0, 87.0, [51, 24], 12 h 16 m, 0 h 23 m	NA
CSSSCL(kmers)	77.0, 76.0, [254, 4], 1 h 7 m, 0 h 4 m	85.0, 86.0, [62, 50], 1 h 50 m, 0 h 19 m	NA
CSSSCL(blast)	92.0, 90.0, [3390, 4], 0 h 31 m, 0 h 0.3 m	89.0, 89.0, [591, 24], 7 h 27 m, 0 h 2 m	95.0, 88.0, [2500, 12], 2 h 7 m, 0 h 4 m
NBC	83.0, 77.0[0.9, 0.04], 0 h 18 m, 18 h 30 m	NA*	77.8, 77.8, [3, NA], NA
Kraken	65.0, 45.0, [686, 13], 0 h 4 m, 0 h 1.5 m	91.0, 82.0, [204, 50], 4 h 3 m, 0 h 6 m	94.7, 73.5, [892 472, 70], NA

In the case of the Bacterial dataset I (full length bacterial sequences), we do not present the results for the NBC and the CSSSCL (but only when the compression measure is included) classifiers due to the very long run time (>4 weeks, marked with NA*), in the case of the Bacterial dataset II (short reads) the CSSSCL program selects only the blast-based similarity measure, since kmer and compression based measures are eliminated (marked with NA) during the optimization phase. In the table, gp/min indicates genomes processed per minute, rp/min indicates reads processed per minute, RAM indicates the maximum RAM usage in GB, time_db indicates the time to process/train the reference database and time_cl to classify sequences in the test set—after the reference database has been processed. The viral dataset was run on a 16 core AMD 64-bit processor with 16 GB of RAM, while the Bacterial datasets were run on a 16 core AMD 64-bit processor with 100 GB of RAM (see also the Supplementary Data for parameter value settings used to run the algorithms).

## 4 Conclusion

We propose a new Python package called CSSSCL to facilitate the accurate taxonomic classification of long and short DNA sequences. By using three new datasets, we confirm the results of our previous findings and show that the implementation of our model presented in Borozan *et* *al.* (2015) is correct and capable of classifying both viral and bacterial sequences with high precision and recall and within a reasonable time frame. Future work will include assessing the performance of additional similarity measures that could be added to the CSSSCL package.

## Supplementary Material

Supplementary Data
