# Supplementary material for: CSSSCL: a python package that uses combined sequence similarity scores for accurate taxonomic classification of long and short sequence reads
Source: Bioinformatics. 2015 Oct 9;32(3):453–5. doi: 10.1093/bioinformatics/btv587 (PMC4734043; doi:10.1093/bioinformatics/btv587)
Supplement: Supplementary Data [file supp_32_3_453__index.html]

CSSSCL: a python package that uses combined sequence similarity scores for accurate taxonomic classification of long and short sequence reads — CSSSCL: a python package that uses combined sequence similarity scores for accurate taxonomic classification of long and short sequence reads — Supplementary Data 

# CSSSCL: a python package that uses combined sequence similarity scores for accurate taxonomic classification of long and short sequence reads

## Supplementary Data

files

- Supplementary Data - pdf file
