# Supplementary material for: CSSSCL: a python package that uses combined sequence similarity scores for accurate taxonomic classification of long and short sequence reads
Source: Bioinformatics. 2015 Oct 9;32(3):453–5. doi: 10.1093/bioinformatics/btv587 (PMC4734043; doi:10.1093/bioinformatics/btv587)
Supplement: Supplementary Data [file supp_btv587_supplementary_data.pdf]

Supplementary Data - CSSSCL: a python package that uses Combined Sequence Similarity Scores for accurate taxonomic CLassification of long and short sequence reads.

Ivan Borozan and Vincent Ferretti

June 2015

0.1 Set of parameters used to run different models/algorithms

CSSSCL :

Viral:
——-
To build the database:
>cssscl build_dbs -btax -c -blast -nt 16 /train_test_viral_full_data/TRAIN.fa /taxon/
To classify:
>cssscl classify -c -blast blastn -tax genus -nt 16 /train_test_viral_full_data/TEST_full.fa /train_test_viral_full_data/
Bacterial I:
————-
To build the database:
>cssscl build_dbs -btax -blast -nt 16 -kmin 4 /bacterial1/TRAIN.fa /taxon/
To classify:
>cssscl classify -blast megablast -num_alignments 1 -nt 16 -tax genus /bacterial1/TEST.fa /bacterial1/
Bacterial II:
————–
To build the database:
>cssscl build_dbs -btax -blast -nt 16 -kmeroff /bacterial2/bacterial_genomes.fna /taxon/
To classify:
>cssscl classify -blast blastn -num_alignments 1 -kmeroff -nt 16 -tax genus /bacterial2/MiSeq_accuracy.fa /bacterial2/

Using the same datasets as above the NBC and Kraken algorithms were used with the following parameters:

NBC:
To build the database:
>countncbi genomes_training_directory 15
To score:
>score -a TEST.fa -r 15 -j genomes_training_directory
Kraken:
To build the database:
>kraken-build –add-to-library TRAIN.fa –db genomeDB
>kraken-build –build –db genomeDB
To run Kraken:
>kraken –threads 16 –preload –db genomeDB TEST.fa –output results_kraken.txt
